# A challenging management of hemophilia B patient with inhibitors undergoing major orthopedic surgeries in a resource‐constrained country

**DOI:** 10.1002/ccr3.3308

**Published:** 2020-09-15

**Authors:** Wafaa Matrane, Afak Nsiri, Mohamed Rafai, Fatima Midmani, Nada Boughaza, Siham Cherkaoui, Meryem Qachouh, Nisrine Khoubila

**Affiliations:** ^1^ Hematology and Pediatric Oncology Department 20 Aout 1953 Hospital Casablanca Morocco; ^2^ Anesthesiology department University Hospital Center Ibn Rochd Casablanca Morocco; ^3^ Orthopedic traumatology department University Hospital Center Ibn Rochd Casablanca Morocco; ^4^ Physiotherapy department University Hospital Center Ibn Rochd Casablanca Morocco

**Keywords:** hematology, orthopedic

## Abstract

In this paper, we report a life‐threatening condition and relate our experience in managing a hemophilia B patient who required three surgical procedures, highlighting the difficulties we encountered in our setting and propose some tangible.

## Graphical abstract

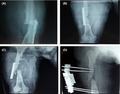

## 1 INTRODUCTION

Hemophilia is a serious lifelong bleeding disorder, affecting 1 in 10 000 males in the world.^1^ Eighty percent of individuals with severe hemophilia live in developing countries, with very limited healthcare resources.^2^ According to WHO estimates, more than 3000 people in Morocco are expected to be affected by hemophilia, but only 1115 cases have been identified in 2011 according to the Moroccan hemophilia patient association. The treatment is mostly limited to episodic therapy and low‐dose prophylaxis at a dose of 50 IU/kg, twice weekly for the majority of patients. Costs vary between 1000 and 1300 euro per month for on demand treatment and around 2500 euro for prophylaxis.^3^

Management of hemophilia is complex and can become more challenging if complicated by development of inhibitory antibodies to coagulation factor VIII or IX.^4^ Inhibitors are much less frequently encountered in hemophilia B patients, occurring in <5% of affected subjects,^5^ and remain high responding in more than 80% of these patients.^6^

Generally and in the presence of inhibitors, standard FVIII or FIX replacement therapy become ineffective, and this is consequently associated with an increase risk of morbidity and mortality, as well as increase in care cost.^7^, ^8^

FIX inhibitor patients differ from those with FVIII inhibitors in several aspects, including the rare occurrence of inhibitors and limited clinical and therapeutic data. The induction of immune tolerance (ITI) is known to be less effective in hemophilia B, and patients may develop allergy or anaphylaxis to FIX concentrates and nephrotic syndrome in conjunction with inhibitor development.

Although elective surgery may improve quality of life for this category of patients, until recently, surgical interventions in patients with hemophilia and inhibitors have been limited to emergency situations and have generally been deferred as long as possible due to the high risk of peri‐operative bleeding and difficulties in hemostatic control.^9^ At present, distinct treatment options exist allowing effective procedures to be safely accomplished in patients with inhibitors.^10^, ^11^

In this paper, we report our experience in managing a hemophilia B patient who required three surgical procedures, highlighting the difficulties we encountered in our setting and propose some tangible.

## 2 CASE REPORT

**FIGURE 1:**
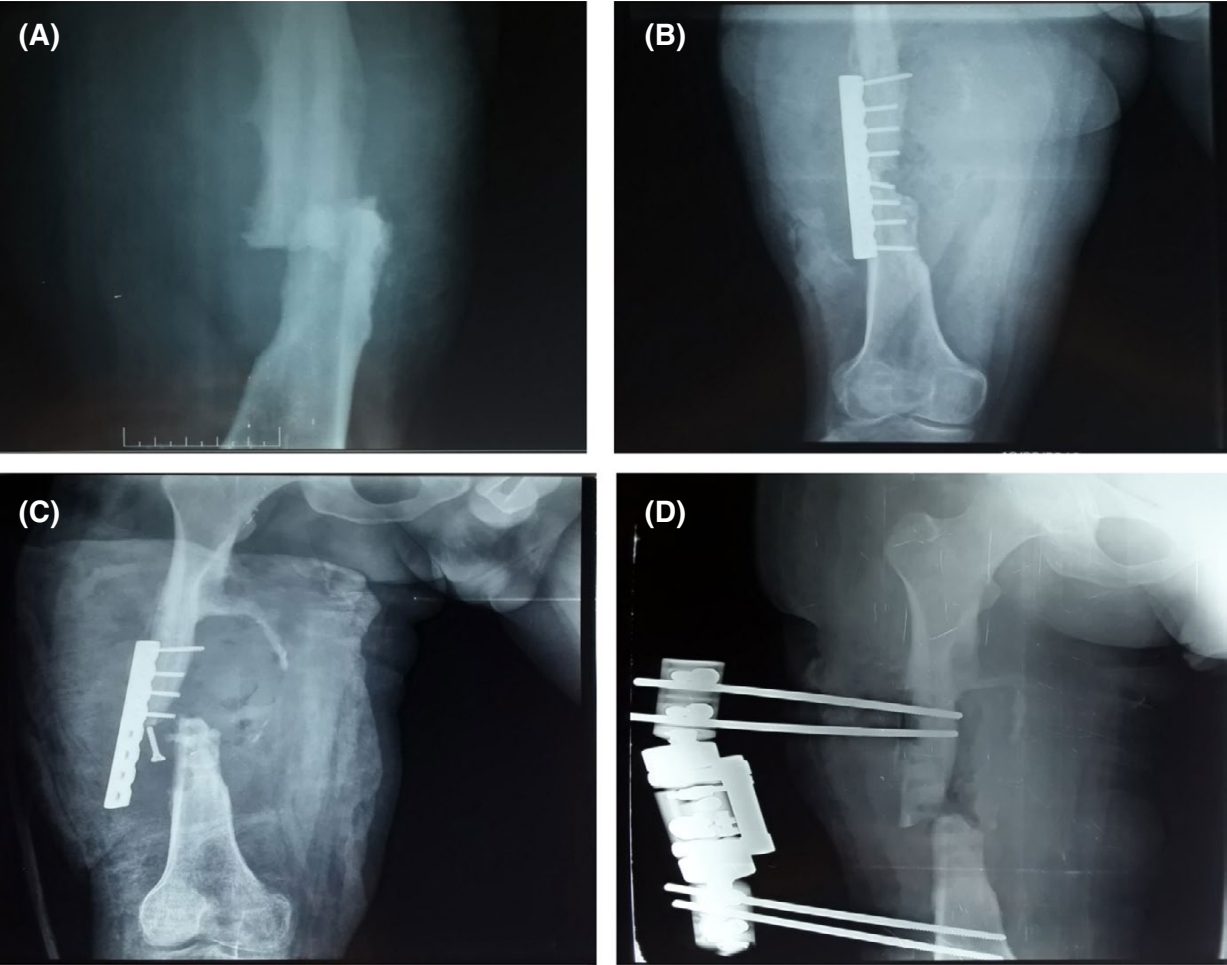
A, Anteroposterior (AP) radiograph demonstrating a comminuted femoral shaft fracture in a 37‐y‐old male patient with hemophilia B. B, Postoperative radiograph of the femur after plate fixation. C, AP radiograph of the femur at postoperative 1 mo demonstrating a nonunion of the femoral shaft and displacement of osteosynthesis material. D, AP radiograph of the femur during a period of short supply in bypassing agents demonstrating a nonunion of the femoral shaft after external fixator

We report the case of 37‐year‐old man, followed since age of 5 years old for moderate hemophilia B with a minor bleeding tendency phenotype and near‐normal musculoskeletal development and no family history of inhibitors. His treatment consisted of episodic treatment (1 or 2 times per year) with recombinant or plasma‐derived FIX concentrates. In July 2018, the patient consulted the department of hematology‐20 Aout Hospital, Casablanca with a comminuted femoral shaft fracture following a traffic accident. On arrival, the patient was conscious with stable vital signs. Physical examination revealed a functional impotence of left lower extremity. Standard X‐ray of the extremity showed a displaced mid‐diaphyseal fracture of left femur (Figure 1). Routine blood tests on admission showed the following results: prothrombin time (PT) of 97%, activated partial thromboplastin time (aPTT) of 132 seconds, fibrinogen of 200 mg/dL, FIX = 1%, absence of inhibitors, hemoglobin (Hb) of 13.5 g/dL, hematocrit of 20,4%, white blood cell of 8500/μL, and platelet of 145 (10^3^/μL). A dynamic compression plate (DCP) osteosynthesis was then performed on the 2th day after injury.

On the day of surgery, recombinant FIX concentrate was administered at a dose of 80 UI/Kg/12 h to maintain activity within the normal physiological coagulation level from day 0 to 2nd day, followed by 40 IU/kg/12 h on day 3, 30 IU/kg/12 h on day 4, and 20 IU/kg/12 h throughout the residual period and until wound healing (16th day).

The surgery was successfully performed, and postoperative bleeding was adequately controlled. The patient was successfully discharged after surgical wound healing and without any sign of bleeding or infection and a Hb level of 12 g/dL on day 18.

**FIGURE 2:**
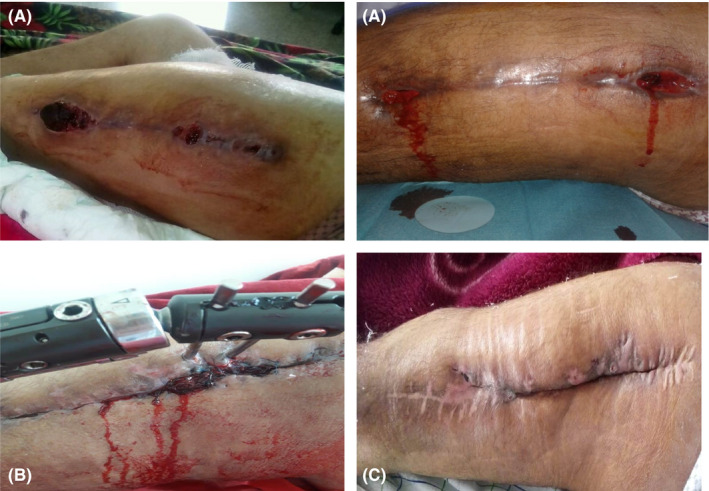
A, At postoperative 1 mo of DCP plate osteosynthesis: hematoma, active bleeding through wound dehiscence. B, Hematoma development after bypassing agents shortage. C, Image of left leg after external fixator removal

At postoperative 1 month, the patient presented with a large hematoma, active bleeding through wound dehiscence (Figure 2), localized infection, generalized pallor, and a Hb level of 4 g/dL. While performing a fluid infusion and blood transfusion (six units of packed red blood cells over 3 days), we conducted femur radiography that demonstrated a nonunion of the femoral shaft and displacement of osteosynthesis material (Figure 1).

Subsequently, recombinant FIX concentrate (50 IU/Kg/12 h) and intravenous antibiotics in response to results of bacteriological samples (Imipenem + Aminoglycoside) were administered. Bleeding persisted despite adequate treatment. Screening tests for antibodies were then carried out, demonstrating high‐titer inhibitors (32 BU). At the point, the patient had less than 50 exposure days of factor during his life. Due to bypassing agents shortage in our context, the patient was suboptimally managed with daily low doses of rFVIIa (NovoSeven^®^ 95 μg/kg/24 h) during 3 months. The evolution was somewhat sufficient, and bleeding and infection were controlled; however, device has exteriorized through the wound. Consequently, osteosynthesis material removal and Orthofix external fixator application was scheduled on December 2018. On the day of the planned surgery, the patient received a bolus infusion of 120 μg/kg of rFVIIa 15 minutes prior to incision and followed by 95 μg/kg every 3 hours. The protocol and evolution of the patient during the peri‐operative period are detailed below:

On day 1, 95 μg/kg of rFVIIa was administered every 4 hours, increased to every 6 hours on day 2 and every 8 hours until day 10. The procedure itself lasted 3 hours, and 45 minutes later, it was complicated by the occurrence of a hemorrhagic shock. The patient was immediately supplied with Noradrenalin (3.0 mg/h), a rFVIIa bolus (120 μg/kg), tranexamic acid (15 mg/kg, every 8 hours), and transfusion of 4 units of packed red blood cells, four platelet units, and five units of fresh frozen plasma (FFP). After transfer and stabilization in the intensive care unit, the Noradrenalin was stopped and the patient was extubated on 2nd postoperative day and transfused with additional three units of red blood cells and 4 FFP, followed by transfer to the Hematology department on day 6. Due to nonavailability of rFVIIa at that point in time, the patient was treated with the available amount of Feiba* (70 IU/Kg/8 h) during a period of 1 month. Immunosuppressive therapy (Rituximab) was then started at the dose of 375 mg/m^2^/week for 4 weeks associated with a low‐dose ITI regimen (50IU/Kg/48 h). The evolution thereafter was favorable followed by a negative FIX inhibitors screening 3 months later.

In December 2019, the patient underwent a 3rd intervention for external fixator removal covered by extended half‐life recombinant FIX, which took place without significant complications and an uneventful postoperative courses. At present, inhibitors titer remains negative at the 3‐month periodic screenings, and the patient is still receiving a low‐dose ITI regimen and regular physiotherapy sessions.

## 3 DISCUSSION

Like other developing countries, Morocco has a geographic disparity in economic and healthcare development, particularly between rural and urban regions. Despite the provided efforts and the increased availability of clotting factor concentrates (CFCs), the existent healthcare delivery system is still not able to fully satisfy all needs of the hemophilia population and therapy remains a serious issue for individuals who have no medical insurance.

Providing hemophilia care in developing countries is a challenge, and patients still suffer from crippling arthropathies, joint damage, and muscle atrophy.^12^

Difficulties may differ from one country to another but they all share the same salient, multifaceted challenges. In most of these countries, hemophilia is not a basic health priority and the healthcare infrastructure is often inadequate, coupled to the low number of human resources dedicated to hemophilia care and the lack of involvement of health insurance services.^13^

Additionally, the limited access to CFC and to bypassing agents, hemophilia care is even more challenging for patients who require surgical interventions, which is often accompanied by the need for additional funding for CFCs, and is associated with additional morbidity due to inhibitor development.^14^

Development of inhibitors remains the most serious treatment‐related complication in hemophilia worldwide, requiring appropriate professional expertise, diagnostic tools, access, and the availability of inhibitor treatment.^1^ Currently, various therapeutic options exist for patients with inhibitors and have been utilized in several surgical interventions in many reports.^9^ Bypassing agents, such as recombinant activated factor VIIa (rFVIIa; NovoSeven) and plasma‐derived activated prothrombin complex concentrates (aPCC; FEIBA), are considered as front‐line therapies for acute bleeding episodes and surgical prophylaxis in hemophilia A and B patients with inhibitors.^15^

Several solutions can be proposed including the promotion of general healthcare infrastructure, fund‐raising, rationalized use of CFCs, adequate utilization of management modalities (such as antifibrinolytic agents and physiotherapy), and the use of prosthetic measures. Patient education remains another serious challenge, requiring careful implementation in countries where illiteracy rates are still high.^16^

These approaches are useful in the majority of resource‐constrained countries. Meanwhile, with the humanitarian aid program of the World Federation of Hemophilia, and governmental efforts to ensure sufficient quantity of CFCs and bypassing agents to meet the needs of this population, all developing countries are gradually witnessing a significant improvement in hemophilia management skills and patient care.

The management of this case was quite difficult given our current context in Morocco. The patient's vital prognosis was compromised several times during the fracture management and the medical team encountered major problems related to the limited availability of bypassing agents and to the occurrence of complications including severe bleeding with hypovolemia, infection, release of wires, and displacement of osteosynthesis material.

Through a multidisciplinary teamwork (hematologist, surgeon, anesthesiologist, and biologist), an appropriate professional expertise and close monitoring, the medical team has succeeded in saving the patient's life and in preserving the functional prognosis of his lower limb despite all difficulties and challenges.

Bleeding and infection management consisted in the use of high doses of antifibrinolytic agents with low levels of bypassing agents to control bleeding to secure hemostasis during surgical procedures, accompanied by fluid resuscitation, transfusion, and antibiotic therapy adapted to the results of bacteriological samples.

In a context where are necessary resources are available, would have permitted us to follow the international guidelines and administer the recommended doses of bypassing agents for our patient, which could have prevented the occurrence of hemorrhagic and orthopedic complications.

Multiple studies have evaluated and reported on the efficacy and the safety of external fixators for management of fractures in patients with hemophilia, despite the possible occurrence of complications including bleeding, infection, and release of wires.^17^ Based on these data, the external fixation remains the best treatment method adapted to the situation of our patient, which raises questions about our initial decision to use internal plating in such a case.

The other basic principle of the treatment besides bleeding management was the eradication of the antibodies through an immunosuppressive therapy followed by low‐dose ITI. ITI is still the only proven and effective approach to eradicate inhibitors.^18^ However, data on the practice of this approach in hemophilia B are limited and reports involving large series in this patient population are lacking.

Induction immune tolerance is a rarely attempted in patients with hemophilia B due to lack of experience of its use and the risk of adverse effects such as allergic reactions and nephrotic syndrome.^18^ Several optimized ITI regimens have been reported with variable outcomes. This therapy is successful in about 60%‐70% of hemophilia A patients but only in 30% in hemophilia B subjects.^5^ Otherwise, a successful use of immunosuppressive agents (Rituximab) as an adjuvant therapy to ITI has been the subject of a few publications.^19^ Indeed, successful tolerization was achieved in our case using low‐dose regimen ITI without the occurrence of any adverse reactions. We were unable to initiate a high dose ITI due to the unmanageable higher associate cost of treatment.

This report provides an opportunity to share our local experience and how with a lower effort and a modest financial undertaking these severe complications can be successfully managed. Thus, it is important that we keep reporting and sharing our cases, experiences, and findings with the medical community to promote and adapt patient management.

## 4 CONCLUSION

Hemophilia care in Morocco is still confronted by many challenges, including the limited number of treatment centers and a short supply of replacement and inhibitor therapy. Despite these constraints, the World Federation of Hemophilia, National Hemophilia Program, and hemophilia professional health care provide community in collaboration with patient associations engage in great efforts to deliver optimal hemophilia care in all regions of Morocco.

## CONFLICT OF INTEREST

The authors declare that there is no conflict of interest.

## AUTHORS CONTRIBUTIONS

WM: contributed to drafting of manuscript. NK, MR, AN, FM, NB, SC, and MQ: contributed to patient management and follow‐up. NK: contributed to critical revision, conception, and final approval.

## ETHICAL APPROVAL

The patient gave informed written consent to publish the identified information and clinical and radiographic images.
